# The Impact of the COVID-19 Pandemic on Ophthalmology Residency Training

**DOI:** 10.18502/jovr.v16i2.9102

**Published:** 2021-04-29

**Authors:** Naveed Nilforushan, Navid Abolfathzadeh

**Affiliations:** ^1^Eye Research Center, The Five Senses Institute, Rassoul Akram Hospital, Iran University of Medical Sciences, Tehran, Iran; ^2^Department of Ophthalmology, Faculty of Medicine, Iran University of Medical Sciences, Tehran, Iran

Dear Editor,

The outbreak of Coronavirus Disease-2019 (COVID-19) has impacted different aspects of life for people all around the world. Students at different levels of education are among those who have been significantly affected by this pandemic. As a higher level of education, ophthalmology residency is a top specialty in different parts of the world including Iran. Ophthalmology residents are expected to learn highly technical surgeries and acquire knowledge throughout their training period. However, during the past year, residency programs have been negatively impacted by this crisis worldwide.^[1,2,3,4]^

Major reasons for this detrimental effect are restriction and suspension of clinical activities and elective surgeries, cancellation of conventional lecturing sessions and educational courses, periodic lockdown of cities, persistent psychological and emotional stress on both trainers and trainees as a consequence of close contact with patients, in addition to loss of colleagues, family members or friends. Moreover, in order to reducing the chance of exposure to infection, the patients themselves prefer to stay at home instead of seeking ophthalmic examinations at university-based centers and especially general hospitals, unless some urgent or emergent situations happen.

Overall, ophthalmology residents have had significant reduction in their clinical and surgical opportunities and are less motivated in their daily routine schedules.^[1]^ Cataract, strabismus, pterygium, lid, and nasolacrimal duct surgeries are among the mandatory group of surgeries in the training curriculum for ophthalmology which are mostly categorized as elective surgeries. On the other hand, urgent cases scheduled for surgery during the pandemic are usually sophisticated ones including vitreoretinal and glaucoma cases that need to be handled by experienced surgeons including fellows and/or attending physicians. Even in the pre-COVID-19 era, direct involvement in such cases was mostly limited and far from the general scope of the residency curriculum. Therefore, the decision to involve residents in such difficult surgical cases in current practice raises multiple ethical dilemmas in the operating room. To involve residents in such cases, we usually prefer general anesthesia instead of routine topical and/or local anesthesia. This leads to prolongation of patient stay at the hospital and also higher workload for the anesthesiology team which can increase the risk of COVID-19 infection for the patient and the medical staff. On the other hand, not including residents in the procedures can result in graduation of less experienced surgeons in future. Therefore, each decision would be a great challenge for the mentors and the heads of ophthalmology departments.

It is expected that the social and economic impacts of this crisis will persist for the next few years and that health systems may face other similar unpredictable crises in the future. Therefore, health authorities should facilitate the incorporation of innovative ways of surgical training such as simulation-based or wet lab-based training to fill this educational gap. We understand that it will take time and need more efforts to reprogram the educational curriculum for ophthalmology residency in the COVID-19 pandemic. In addition, we know that moving toward such innovative ways will impose a significant economic burden on the health system especially in developing countries which are already under stress for funding sources related to the COVID-19 pandemic; therefore, this approach may not be among the priorities of health policymakers.

It is good to say that, besides the negative and tragic impacts of this pandemic, new opportunities have emerged in the field of education which will definitely change our practice in future. There are plenty of affordable e-learning facilities for improving residency education including offline surgical movie clips, online virtual grand-rounds like web-based case presentations and journal clubs, webcasts and online national and international webinars. These positive changes have happened mostly after the COVID-19 pandemic, in which online webinars are the most widespread ones. They let large numbers of ophthalmologists from all over the world to be involved, discuss, and learn simultaneously. Such online gatherings will undoubtedly remain and continue even after the pandemic ceases. However, one should keep in mind that developing countries face obstacles in accessing online programs including internet connectivity and speed, in addition to the cost.

These new circumstances have also changed our communication with patients and made telemedicine-based management more prominent. Nowadays, patients can easily contact their physicians and send them information, images, and tests using smartphones. All of these may lead to safer and more cost-effective patient visits. Telemedicine and its applications can be a part of the education during these hard times.

Last but not the least, it is important to perform and validate residency examination and evaluations in a standardized and uniform electronic format. Attention should also be paid to necessary changes in the national curriculum of ophthalmology residency education, to prepare residents with non-surgical but fundamental skills such as crisis management and teamwork.^[5]^ Additionally, as a priority, we must ensure that our trainees are and will be safe and healthy during their education period, therefore all necessary protective equipment and measures should be provided to fulfill this goal.

##  Financial Support and Sponsorship

Nil.

##  Conflicts of interest

There are no conflicts of interest.
